# Supplementary material for: Mindful Eating Questionnaire: Validation and Reliability in Romanian Adults
Source: Int J Environ Res Public Health. 2022 Aug 24;19(17):10517. doi: 10.3390/ijerph191710517 (PMC9518582; doi:10.3390/ijerph191710517)
Supplement: Supplementary file 1 [file ijerph-19-10517-s001.zip › ijerph-1853870-supplementarypdf.pdf]

Supplementary Material

Table S1. Mindful Eating Questionnaire–Romanian version.

Questions	Intrebari
1. I eat so quickly that I don't taste what I'm eating.	1. Mănânc așa de repede încât nu simt ceea ce mănânc.
2. When I eat at "all you can eat" buffets, I tend to overeat.	2. Când mănânc la "bufet suedez" am tendința să îmi pun în exces.
3. At a party where there is a lot of good food, I notice when it makes me want to eat more food than I should.	3. La o petrecere la care se mănâncă multă mâncare bună, observ când îmi vine să mănânc mai multă mâncare decât ar trebui.
4. I recognize when food advertisements make me want to eat.	4. Conștientizez când reclamele la alimente mă fac să vreau să manânc.
5. When a restaurant portion is too large, I stop eating when I'm full.	5. Când o porție de la restaurant este prea mare, mă opresc din mâncat când sunt sătul.
6. My thoughts tend to wander while I am eating.	6. Gândurile mele tind să rătăcească în timp ce mănânc.
7. When I'm eating one of my favorite foods, I don't recognize when I've had enough.	7. Când mănânc unul dintre alimentele mele preferate, nu știu când să mă opresc.
8. I notice when just going into a movie theater makes me want to eat candy or popcorn.	8. Îmi dau seama că dacă mă aflu într-un cinematograf îmi vine să mănânc popcorn sau dulciuri.
9. If it doesn't cost much more, I get the larger size food or drink regardless of how hungry I feel.	9. Dacă nu costă mult în plus, prefer porția mare de mâncare sau băutură, indiferent de cât de foame îmi este.
10. I notice when there are subtle flavors in the foods I eat.	10. Îmi dau seama când există arome subtile în alimentele pe care le consum.
11. If there are leftovers that I like, I take a second helping even though I'm full.	11. Dacă de la o masă rămâne mâncare care îmi place, iau o a doua porție, chiar dacă sunt sătul(ă).
12. When eating a pleasant meal, I notice if it makes me feel relaxed.	12. Când mănânc mâncare care îmi place, observ că mă face să mă simt relaxat(ă).
13. I snack without noticing that I am eating.	13. Iau gustări fără să realizez că mănânc.
14. When I eat a big meal, I notice if it makes me feel heavy or sluggish.	14. Când mănânc o masă copioasă, observ dacă mă face să mă simt greu sau moleșit.
15. I stop eating when I'm full even when eating something I love.	15. Mă opresc din mâncat când mă simt sătul(ă), chiar dacă mănânc ceva ce îmi place.
16. I appreciate the way my food looks on my plate.	16. Apreciez modul de prezentare din farfurie.
17. When I'm feeling stressed at work, I'll go find something to eat.	17. Când muncesc și mă simt stresat(ă), merg să găsesc ceva de mâncare.
18. If there's good food at a party, I'll continue eating even after I'm full.	18. Dacă există mâncare bună la o petrecere, voi continua să mănânc chiar și după ce sunt sătul(ă).
19. When I'm sad, I eat to feel better.	19. Când sunt trist, mănânc ca să mă simt mai bine.
20. I notice when foods and drinks are too sweet.	20. Îmi dau seama când mâncarea sau băutura sunt prea dulci.
21. Before I eat I take a moment to appreciate the colors and smells of my food.	21. Înainte să mănânc, stau un pic să evaluez culorile și mirosul mâncării.

22. I taste every bite of food that I eat.	22. Savurez fiecare îmbucătură din mâncare.
23. I recognize when I'm eating and not hungry.	23. Îmi dau seama că mănânc, deși nu îmi este foame.
24. I notice when I'm eating from a dish of candy just because it's there.	24. Îmi dau seama când mănânc dintr-un bol cu bomboane doar pentru că sunt acolo.
25. When I'm at a restaurant, I can tell when the portion I've been served is too large for me.	25. Când sunt la restaurant, îmi dau seama când porția servită este prea mare pentru mine.
26. I notice when the food I eat affects my emotional state.	26. Îmi dau seama când mâncarea îmi schimbă starea emoțională.
27. I have trouble not eating ice cream, cookies, or chips if they're around the house.	27. Nu mă pot abține din a mânca înghețată, prăjituri sau chipsuri dacă le găsesc în casă.
28. I think about things I need to do while I am eating.	28. Când mănânc, mă gândesc la lucruri pe care trebuie să le fac.
